# Supplementary material for: Spatial Neglect in Stroke: Identification, Disease Process and Association with Outcome During Inpatient Rehabilitation
Source: Brain Sci. 2019 Dec 13;9(12):374. doi: 10.3390/brainsci9120374 (PMC6956021; doi:10.3390/brainsci9120374)
Supplement: Supplementary file 1 [file brainsci-09-00374-s001.pdf]

Table S1. Cross tabulation of the presence of each NIHSS item (>0) by Spatial Neglect.

NIHSS Item	Present?	Q11 Spatial Neglect Frequency (Row%) Missing	No(=0)	Yes(>0)	Total
Q1a Level of Consciousness	No (=0)	2126 (2.8)	56,537 (73.8)	17,922 (23.4)	76,585
	Yes (>0)	4115 (22.5)	5411 (29.5)	8794 (48.0)	18,320
Q1b LOC Questions	Missing	3558 (81.7)	424 (9.7)	374 (8.6)	4356
	No (=0)	734 (1.3)	45,100 (79.7)	10,745 (19.0)	56,579
	Yes (>0)	1949 (5.7)	16,424 (48.3)	15,597 (45.9)	33,970
Q1c LOC Commands	Missing	3583 (86.0)	321 (7.7)	261 (6.3)	4165
	No (=0)	1076 (1.5)	53,477 (76.0)	15,784 (22.4)	70,337
	Yes (>0)	1582 (7.8)	8150 (39.9)	10,671 (52.3)	20,403
Q2 Best Gaze	Missing	4398 (77.8)	597 (10.6)	660 (11.7)	5655
	No (=0)	1207 (1.7)	56,106 (80.3)	12,594 (18.0)	69,907
	Yes (>0)	636 (3.3)	5245 (27.1)	13,462 (69.6)	19,343
Q3 Visual	Missing	4831 (66.1)	1027 (14.0)	1453 (19.9)	7311
	No (=0)	852 (1.4)	51,634 (84.3)	8791 (14.3)	61,277
	Yes (>0)	558 (2.1)	9287 (35.3)	16,472 (62.6)	26,317
Q4 Facial Palsy	Missing	3561 (90.5)	174 (4.4)	201 (5.1)	3936
	No (=0)	979 (2.4)	33,869 (82.9)	6028 (14.7)	40,876
	Yes (>0)	1701 (3.4)	27,905 (55.7)	20,487 (40.9)	50,093
Q5a Motor Arm (Left)	Missing	3421 (90.4)	197 (5.2)	166 (4.4)	3784
	No (=0)	1547 (2.7)	43,390 (75.4)	12,612 (21.9)	57,549
	Yes (>0)	1273 (3.8)	18,361 (54.7)	13,938 (41.5)	33,572
Q5b Motor Arm (Right)	Missing	3411 (90.6)	177 (4.7)	177 (4.7)	3765
	No (=0)	1304 (2.2)	43,519 (72.8)	14,942 (25.0)	59,765
	Yes (>0)	1526 (4.9)	18,252 (58.2)	11,597 (37.0)	31,375
Q6a Motor Leg (Left)	Missing	3532 (86.2)	299 (7.3)	266 (6.5)	4097
	No (=0)	1429 (2.5)	44,178 (76.3)	12,315 (21.3)	57,922
	Yes (>0)	1280 (3.9)	17,471 (53.1)	14,135 (43.0)	32,886
Q6b Motor Leg (Right)	Missing	3551 (86.0)	305 (7.4)	273 (6.6)	4129
	No (=0)	1253 (2.1)	44,658 (74.1)	14,326 (23.8)	60,237
	Yes (>0)	1437 (4.7)	16,985 (55.6)	12,117 (39.7)	30,539
Q7 Limb Ataxia	Missing	5131 (59.4)	1342 (15.5)	2159 (25.0)	8632
	No (=0)	818 (1.2)	48,275 (70.3)	19,542 (28.5)	68,635
	Yes (>0)	292 (1.7)	12,331 (69.9)	5015 (28.4)	17,638
Q8 Sensory	Missing	5218 (69.8)	863 (11.5)	1398 (18.7)	7479
	No (=0)	606 (1.0)	48,156 (83.3)	9043 (15.6)	57,805
	Yes (>0)	417 (1.4)	12,929 (43.6)	16,275 (54.9)	29,621
Q9 Best Language	Missing	3920 (89.3)	190 (4.3)	282 (6.4)	4392
	No (=0)	501 (0.9)	41,861 (79.0)	10,614 (20.0)	52,976
	Yes (>0)	1820 (4.8)	19,897 (53.0)	15,820 (42.1)	37,537
Q10 Dysarthria	Missing	4372 (80.2)	306 (5.6)	774 (14.2)	5452
	No (=0)	500 (1.2)	35,157 (81.5)	7466 (17.3)	43,123
	Yes (>0)	1369 (3.0)	26,485 (57.2)	18,476 (39.9)	46,330
Total		6241 (6.6)	61,948 (65.3)	26,716 (28.2)	94,905

LOC compulsory resulting in no missing data.

Table S2. Combined combinations of NIHSS Motor Impairment items (Left/Right Leg and Arm) by Spatial Neglect.

Combined Recorded Outcomes of NIHSS Item Motor Q5a-6b (Arm/Leg/Left/Right)	Q11 Spatial Neglect (Frequency (Row%))			
	**Missing**	**No (=0)**	**Yes (>0)**	**Total**
None	3795 (12.5)	23591 (77.9)	2903 (9.6)	30,289
LA	128 (2.6)	3746 (75.1)	1116 (22.4)	4990
RA	152 (3.1)	3967 (80.4)	814 (16.5)	4933
LL	35 (1.8)	1651 (83.1)	300 (15.1)	1986
RL	30 (1.7)	1488 (83.9)	256 (14.4)	1774
LA LL	612 (2.9)	11474 (54.1)	9139 (43.1)	21,225
RA RL	784 (4.1)	11114 (58.4)	7144 (37.5)	19,042
LA LL RA	25 (8.2)	141 (46.1)	140 (45.8)	306
RA RL LA	24 (8.0)	137 (45.8)	138 (46.2)	299
LA LL RL	66 (3.3)	840 (42.5)	1071 (54.2)	1977
RA RL LL	114 (5.5)	911 (43.9)	1050 (50.6)	2075
LA RA	38 (11.2)	223 (65.6)	79 (23.2)	340
LL RL	39 (4.2)	695 (74.2)	203 (21.7)	937
LA RL	10 (2.8)	211 (59.9)	131 (37.2)	352
RA LL	19 (6.4)	170 (57.2)	108 (36.4)	297
All 4	370 (9.1)	1589 (38.9)	2124 (52.0)	4083
Total	6241 (6.6)	61948 (65.3)	26716 (28.2)	94,905

RL = Right Leg, LL = Left Leg, RA = Right Arm, LA = Left Arm. In each case score>0. Note, if score = 0 or missing, motor impairment deemed not present.
